# Supplementary material for: The ecological footprint of physicians: A survey of physicians in Canada, India, and USA
Source: PLoS One. 2023 Sep 12;18(9):e0291501. doi: 10.1371/journal.pone.0291501 (PMC10497187; doi:10.1371/journal.pone.0291501)
Supplement: S1 Appendix — (DOCX) [file pone.0291501.s001.docx]

**Appendix**

**Supplementary Table-** Supplementary Survey Results

|  | **All respondents (n=162)** | **India  (n=49)** | **Canada  (n=57)** | **USA  (n=56)** |
| --- | --- | --- | --- | --- |
| **Number of km/mi traveled by gasoline/petrol/diesel-based vehicles per week** |  |  |  |  |
| 0 km (0 mi) | 9 (6%) | 3 (6%) | 0 | 6 (11%) |
| <100 km (<62 mi) | 102 (63%) | 30 (61%) | 32 (56%) | 40 (71%) |
| 100-200 km (62-124 mi) | 35 (22%) | 12 (24.5%) | 16 (28%) | 7 (12.5%) |
| 200-300 km (124-186 mi) | 9 (6%) | 2 (4%) | 6 (10.5%) | 1 (2%) |
| 300+ km (186+ mi) | 7 (4%) | 2 (4%) | 3 (5%) | 2 (4%) |
|  |  |  |  |  |
| **Mean number of people in household** | 2.8 | 4.1 | 2.4 | 2.2 |
|  |  |  |  |  |
| **Own a second home** | 10 (6%) | 3 (6%) | 3 (5%) | 4 (7%) |
|  |  |  |  |  |
| **Number of cars in household** |  |  |  |  |
| 0 | 20 (12%) | 10 (20%) | 4 (7%) | 6 (10%) |
| 1 | 64 (40%) | 22 (45%) | 22 (39%) | 20 (36%) |
| >1 | 78 (48%) | 17 (35%) | 31 (54%) | 30 (54%) |
|  |  |  |  |  |
| **Own an electric car** | 11 (7%) | 2 (4%) | 2 (3.5%) | 7 (12.5%) |
|  |  |  |  |  |
| **Hours of flying (2019)** |  |  |  |  |
| 0 | 26 (17%) | 14 (33%) | 9 (16%) | 3 (5%) |
| 1-10 hours | 37 (24%) | 16 (37%) | 11 (20%) | 10 (18%) |
| >10 hours | 91 (59%) | 13 (30%) | 36 (64%) | 42 (77%) |
|  |  |  |  |  |
| **Self-comparison of ecological footprint to peers** |  |  |  |  |
| Larger than peers | 9 (6%) | 5 (10%) | 4 (7%) | 0 |
| Comparable to peers | 107 (66%) | 24 (49%) | 41 (72%) | 42 (75%) |
| Smaller than peers | 46 (28%) | 20 (41%) | 12 (21%) | 14 (25%) |

**Survey Questions**

1. CONSENT: I have read the information presented in the information letter about a study being conducted by Dr. Arsha Karbassi and Faramarz Jabbari-Zadeh of McMaster University.  I have had the opportunity to ask questions about my involvement in this study and to receive additional details I requested.  I understand that if I agree to participate in this study, I may withdraw from the study at any time before I submit the survey.  I agree to participate in the study. I have reviewed the information about this study, and I understand that once I click 'submit' at the end of the survey, I have consented to participate. I also understand that once I submit my responses, my data cannot be removed.

a. Yes

b. No

1. What term best describes you?
   1. Medical student
   2. Resident/Fellow
   3. Practicing physician
   4. None of the above
2. What is your age group?
   1. 18-24
   2. 25-34
   3. 35-44
   4. 45-54
   5. 55+
3. Where do you currently live?
   1. Canada
   2. The United States of America
   3. India
4. Which description best fits your place of residence?
   1. Detached house
   2. Detached house with a swimming pool
   3. Semi-detached house/Rowhouse/Townhouse
   4. Multi-storey apartment or condominium
5. Do you own a second home, such as a cabin or a vacation property? (Do NOT include investment properties, for instance, properties that are bought to be rented out).
   1. Yes
   2. No
6. How many people do you live with? Include all children above the age of 2, and all people who have stayed more than 6 months in the house over the past year.
   1. I live alone
   2. 1
   3. 2
   4. 3
   5. 4
   6. 5
   7. 6+
7. What is your predominant mode of transportation to work?
   1. Walking/bicycling
   2. Public transportation (bus, train)
   3. Motorcycle
   4. Car
8. How many cars does your household have?
   1. 0
   2. 1
   3. 2
   4. ≥ 3
9. Do you own an electric car?
   1. Yes
   2. No
10. On average, how far do you travel by gasoline/petrol/diesel-based vehicles per week?
    1. 0 km (0 mi)
    2. < 100 km (< 62 mi)
    3. 100-200 km (62-124 mi)
    4. 200-300 km (124-186 mi)
    5. 300+ km (186+ mi)
11. How many hours do you fly each year? Please reflect on your travel in 2019 (free text).
12. How frequently do you eat animal products (meat and/or dairy)?
    1. Never or rarely
    2. Infrequently (occasional eggs/dairy, no meat)
    3. Vegetarian (frequent eggs/dairy, close to no meat)
    4. Occasionally (meat 1-2 times a week)
    5. Often (meat 3-5 times a week)
    6. Very often (meat daily)
    7. I usually eat meat for ≥ 2 meals a day
13. Compared to 5 years ago, how has your consumption of meat and dairy changed?
    1. Increased a lot
    2. Increased a little
    3. Approximately the same
    4. Decreased a little
    5. Decreased a lot
14. Which of these statements do you agree with the most?
    1. The responsibility for mitigating climate change lies nearly completely with corporations and governments. Individual actions are largely inconsequential, and serve primarily as a distraction.
    2. How each of us individually live, primarily determines how the world is used. It is therefore up to us, as individuals, to adopt practices that are environmentally friendly and that can help us live sustainably.
    3. We need a system change rather than individual change, but you cannot have one without the other. Individual action plays a critical role in achieving structural change.
15. How would you characterize your own ecological footprint compared to peers? Peers are fellow medical students/physicians living in the same country.
    1. Larger than my peers
    2. Comparable to my peers
    3. Smaller than my peers
16. Are you open to reducing your own ecological footprint?
    1. Yes and I have taken steps to reduce my ecological footprint.
    2. Yes, but I have taken few/no steps to reduce my ecological footprint.
    3. No, I am not able to in my current circumstances.
    4. No, I do not think reducing my individual ecological footprint will make a difference.
17. How often does climate change get mentioned during a conversation with colleagues?
    1. Rare or never
    2. 1-2 times per year
    3. 3-6 times per year
    4. 6-12 times per year
    5. > once a month
    6. > once a week
18. How often does climate change get mentioned during a conversation with family/friends?
    1. Rare or never
    2. 1-2 times per year
    3. 3-6 times per year
    4. 6-12 times per year
    5. > once a month
    6. > once a week
