# Supplementary material for: The ecological footprint of physicians: A survey of physicians in Canada, India, and USA
Source: PLoS One. 2023 Sep 12;18(9):e0291501. doi: 10.1371/journal.pone.0291501 (PMC10497187; doi:10.1371/journal.pone.0291501)
Supplement: Attachment — Submitted filename: Response to reviewers.docx [file pone.0291501.s003.docx]

Reviewer #1: Dear Authors,
Congratulations for your interesting research.
The title and the abstract coincide with the content of the paper. Keywords are well-chosen.
The problem and goal of the paper are clearly and correctly formulated.
The manuscript is clear, relevant for the field and presented in a well-structured manner.
To achieve the goal of the paper, the applied research methods can be considered correct.
The paper contains a comprehensive reference list. The literature studies presented should be considered sufficient both as to the proper selection of sources and their quantity. The cited references are current.
The manuscript’s results are reproducible based on the details given in the methods section.
The figures and the tables are appropriate. They properly show the data. They are easy to interpret and understand.

**Response:** Thank you for taking the time to provide a thorough review of our manuscript. We deeply appreciate your comments and encouragement.

Reviewer #2: The work deals with an interesting issue about ecological footprints. In my opinion, this is a timely topic. However, I have a few comments to improve the quality of the article.
- Improve the abstract so that it is an abstract without being broken down into chapters.

**Response:** Thank you for taking the time to review our manuscript, and provide constructive feedback. We have now re-formatted the abstract and removed chapters.

- Please explain how the obtained results relate to the carbon footprint of the inhabitants of the given countries?

**Response:** We have added the following line with a reference in the first paragraph under Discussion,

‘This is consistent with population level data from these countries, with average per capita CO_2_ emissions in USA and Canada nearly 15 tonnes, compared to 2 tonnes in India.’

- Please indicate why this occupational group was studied and not another?
**Response:** The focus on physicians is because of their unique position in society, as they have been staunch advocates for climate change action, but at the same time, may have a high carbon footprint on account of their wealth/income. We elaborated on this in the Introduction, and have added a line in the introduction to clarify this further,

‘However, physicians are usually amongst the wealthiest in most societies (with many in the top 1% globally by income and/or wealth), and medical learners often come from high socioeconomic backgrounds’

- What does high carbon footprint mean? what was the indicator?
**Response:** A low ecological footprint was defined as meat intake ≤2 times per week, living in an apartment or condominium and using public transport, bicycle, motorcycle or walking to work. Any participant that did not meet criteria for low ecological footprint was classified as having a high ecological footprint. We have mentioned this in Results under ‘Ecological Footprint’, and have now also added this in the Methods section.

- It should be clearly emphasized in the work that 96/162 people are students. Is the result statistically correct, referring to the research sample?
**Response:** Thank you for highlighting this important limitation. In the first paragraph under Results, we have mentioned ‘The respondents were primarily young physicians in training, with 122 (75%) of them <35 years of age and 96 (59%) in medical school, residency, or fellowship.’

We have also now added this under Limitations, ‘…the people who chose not to respond to our survey may have differed in important ways from those who did participate, which would lead to a biased sample. For instance, three quarters of our respondents were younger than 35 years of age (with 59% in medical school, residency, or fellowship), so our data may not be adequately representative of older physicians.’

- Please explain how these groups "food", transport, "Climate change talks" relate to the carbon footprint?

**Response:** We categorized total ecological footprint by building, transport, and food sectors, in accordance with IPCC convention for sector-wise emissions. The sub-headings of ‘Beliefs and Attitudes’ and ‘Conversations Around Climate Change’ in Results refers to additional survey questions, and are not a direct source of high ecological footprint.

We have mentioned this under Methods,

‘Participants were surveyed for resource consumption in food, building, and transport sectors, along with changes in consumption patterns over time. We also surveyed beliefs and attitudes towards climate change mitigation.’

- The discussion of the results is correct.
**Response:** Thank you!

- The Conclusions chapter is really a summary, not a conclusion. They should be added and specific research limitations and further research plans of the authors in this subject should be indicated.

**Response:** As suggested, we have modified the Conclusions chapter accordingly. We have also expanded the section on ‘Limitations and Future Research Direction’, adding a line- ‘Future research should aim to recruit older physicians, who may not be adequately sampled by research methods that rely solely on online distribution.’
